# Case Report: A rare cause of vaginal bleeding at Keren Zonal Referral Hospital, Eritrea

**DOI:** 10.12688/f1000research.17453.2

**Published:** 2021-04-08

**Authors:** Million Abraha, Shiden Solomon

**Affiliations:** 1 Fairmed, Bern, 3011, Switzerland; 2 Institute for Social and Preventive Medicine, University of Bern, Bern, 3012, Switzerland; 3 Maurice Wohl Basic and Clinical Neuroscience Institute, King’s College London, London, WC2R 2LS, UK

**Keywords:** Leech, Vaginal bleeding, Anemia, Infestation, Case report

## Abstract

Vaginal bleeding due to leech infestation is a very uncommon but important gynaecological problem. This report presents the case of a 65-year-old woman who presented to Keren Regional Referral Hospital, Eritrea, with vaginal bleeding of two and half weeks duration, dizziness and fatigue. On the day of her admission complete blood count and speculum exam were done and she was diagnosed with anaemia due to cervical leech infestation. Routine speculum exam for vaginal bleeding is recommended in cases with history of holy water or springs visits to prevent unnecessary diagnostic tests and for rapid management.

## Introduction

Leeches, which belong to the phylum Annelida and class Hirudinea, are blood-sucking parasites of most mammals, including humans. Though there are about 650 known species of leech, only a few of them are a threat to human health. People and livestock that walk close by and frequently have access to marsh areas or slow-moving streams and brocks are among the frequent victims of leech infestation ^1^. Moreover, there have been reports of climatic variation associated with leech infestation, where leeches are more frequently found in tropical and subtropical areas ^2^.

Leeches have a long slender body, 5–45mm, with an oral sucker as a mouth and caudal end for movement ^3^. Leeches bite different body sites of victims, which includes the pharynx, larynx, oesophagus, rectum, vagina, urethra and bladder, which have been reported across different publications ^1, 3, 4^.

When leeches become in close contact with human bodies, they tend to attach themselves to the mucosal surfaces and secrete an anticoagulant that leads to excessive bleeding from the attachment site ^2, 5^. If it is not diagnosed as early as possible, this seemingly harmless parasite, could lead to some life threatening anaemia and shock ^2^. In this paper we present a referral case of rare vaginal bleeding due to cervical leech infestation.

## Case presentation

A 65-year-old para IV patient with no history of abortion, presented with vaginal bleeding of 17 days. She was referred to Keren Zonal Referral Hospital, Eritrea, from Adi-tekelezan Health Centre with a diagnosis of vaginitis.

Upon arrival, the general condition of the mother was stable. She had a fresh blood soaking her clothes. The patient reported that she was bleeding intermittently at home followed by big clots of blood. Since the start of the bleeding the patient was feeling dizzy and was becoming fatigued easily. A few days prior to admission to hospital, she was feeling an abnormal moving sensation in her abdomen. The patient stated that previous to her symptoms, she had visited Amne-Tekle Bahri, a holy water nearby, to help her alleviate headache and psychological problems.

Upon thorough examination, the patient had blood pressure of 90/40 mm Hg (normal range 90/60 mm Hg to 120/80 mm Hg), pulse rate 140 beats per minute (normal range, 60 to 100 beats per minute) and respiratory rate 33 per minute (normal range, 12 to 18 breaths per minute). She had pale conjunctiva. The perineal area was stained with blood, the cervix was closed, and the uterus was normal in size and consistency. Speculum examination revealed an actively moving worm attached to the external orifice of the cervix (a similar visual representation can be seen in Tilahun (2015) ^6^ Figure 1). Complete blood count showed a haemoglobin level of 4.6g/dl (normal range, 12 g/dL to 16.0 g/dL) and platelet count of 110,000 cells/microliter (normal range, 150 × 10 ^3^ μL to 400 × 10 ^3^ μL). Abdominal ultrasound finding was normal.

The patient was transfused with two units of blood immediately and the bleeding site was gently washed with saline and a bit of alcohol to facilitate the detachment of the leech. With a lot of care, the leech was removed gently using sterile forceps. After stabilising the vital signs (respiratory rate 17 breaths per minute, blood pressure 110/80 mm Hg, pulse rate 80 beats per minute), the patient was sent home after four days with iron tablets and with advice to take a good preventive measures if she had ever to go to the holy water again. She was also informed to bring the accompanying family members who went with her if they showed any unusual symptoms. Pre-discharge complete blood cell count showed an increased Hg level of 8g/dl and platelet count of 120,000 cells/microliter. The patient was asked to come back after two weeks for follow-up. A speculum exam was done, and the attachment site was completely healed and no active bleeding was observed.

## Discussion

Vaginal bleeding due to leech infestation is a very uncommon complaint in women visiting gynaecological and obstetrics departments ^7– 9^. Vaginal bleeding due to worm infestation could usually be missed if thorough investigations and physical examination are not done accordingly. A speculum exam should be done to see the source of bleeding and possible leeches before entertaining other differential diagnosis, especially if a patient reports visiting water bodies. Great care should be taken in removing the worm from its attachment as the procedure might also cause additional bleeding due to injury from the site of attachment and from the areas around it. It should not be forcefully removed as the jaws of the worm might remain at the bite site which might cause additional bleeding or lead to later malignancies ^10^. Preventive measures such as self-checking after washing in ponds and waterfalls are necessary to minimize infestation and instructions should be given as part of routine health education. An awareness program should be initiated to inform clinicians working in clinics near such sources of water.

## Consent

Written informed consent for the publication of the case report was obtained from the patient.

## Data availability

No data is associated with this article.

## Author information

MA was responsible for the gynaecological management of the patient while based at Keren Zonal Referral Hospital in 2012. SS was the nurse collaborating on this case while based at Halibet Hospital.
