# Supplementary material for: Case Report: A rare cause of vaginal bleeding at Keren Zonal Referral Hospital, Eritrea
Source: F1000Res. 2021 Apr 8;8:37. Originally published 2019 Jan 9. [Version 2] doi: 10.12688/f1000research.17453.2 (PMC8108554; doi:10.12688/f1000research.17453.2)
Supplement: Peer review history

# Peer review: Reviewer response for version 2

**DOI:** 10.5256/f1000research.55704.r82890

**Published:** 2021-05-07

**Authors:** Temesgen Tilahun

**Affiliations:** 1 Department of Obstetrics and Gynecology, Wollega University, Nekemte, Ethiopia

I went through it and found it great. They modified it well. Thus, it can be indexed.

Are enough details provided of any physical examination and diagnostic tests, treatment given and outcomes?

Yes

Is the case presented with sufficient detail to be useful for other practitioners?

Yes

Is sufficient discussion included of the importance of the findings and their relevance to future understanding of disease processes, diagnosis or treatment?

Partly

Is the background of the case’s history and progression described in sufficient detail?

Yes

Reviewer Expertise:

Gynecology.

I confirm that I have read this submission and believe that I have an appropriate level of expertise to confirm that it is of an acceptable scientific standard.

# Peer review: Reviewer response for version 1

**DOI:** 10.5256/f1000research.19087.r52696

**Published:** 2019-08-20

**Authors:** Hossein Zarrinfar

**Affiliations:** 1 Mashhad University of Medical Sciences, Mashhad, Iran

In the manuscript entitled "Case Report: A rare cause of vaginal bleeding at Keren Zonal Referral Hospital, Eritrea", the authors have described vaginal bleeding. This manuscript is interesting and educational, but there is no figure related to the leech, that should be added. By the way, there are some grammatical and spelling errors in English too that should be corrected by an expert.

**Abstract:**

1. “This report presents the case of a 65-year-old woman who presented to Keren Regional Referral Hospital, Eritrea, with vaginal bleeding of two and half weeks duration, dizziness and fatigue. On the day of her admission complete blood cell count and speculum exam were done and she was diagnosed with anaemia due to cervical leech infestation”. The sentences are unclear and need revision.
2. The conclusion should be revised according to study aim again. Hence, “Routine speculum exam for vaginal bleeding is recommended in cases with history of holy water or springs visits to prevent unnecessary diagnostic tests and for rapid management” to be omitted.

**Introduction:**

1. “…more frequently found in tropical and subtropical areas”, this sentence needs another newer reference. Please add this ^1^.
2. “If it is not diagnosed as early …. Anaemia and shock” the sentences are unclear and needs a revision.

**Discussion:**

1. This section should be revised as predisposing factors to leeches in other studies. The conclusion should be revised according to study aim and the results again.

Are enough details provided of any physical examination and diagnostic tests, treatment given and outcomes?

Partly

Is the case presented with sufficient detail to be useful for other practitioners?

Yes

Is sufficient discussion included of the importance of the findings and their relevance to future understanding of disease processes, diagnosis or treatment?

No

Is the background of the case’s history and progression described in sufficient detail?

Partly

Reviewer Expertise:

Microbiology, Mycology.

I confirm that I have read this submission and believe that I have an appropriate level of expertise to confirm that it is of an acceptable scientific standard.

## References

[1] : Unusual vaginal bleeding due to a leech bite in a girl from a tropical area: A case report. *Rev Soc Bras Med Trop*.2019;52: 10.1590/0037-8682-0425-2018 e20180425 10.1590/0037-8682-0425-2018 30994809

# Peer review: Reviewer response for version 1

**DOI:** 10.5256/f1000research.19087.r51366

**Published:** 2019-07-31

**Authors:** Temesgen Tilahun

**Affiliations:** 1 Department of Obstetrics and Gynecology, Wollega University, Nekemte, Ethiopia

**Abstract**

- Line 5 ‘complete blood cell count’ write as 'complete blood count'.

**Case presentation**

1. Good to replace the word ‘mother’ with ‘patient’.
2. Line 2 ‘17 days duration’ write as ‘17 days’.
3. ‘a bit of alcohol to facilitate the detachment of the leech’. It is clear that alcohol isan irritant to the vagina. How did you use it? Is that contraindicated in the vagina? This needs explanation.
4. This patient was appointed for follow up but what happened then?
5. The authors must include a picture of the leech.

**Discussion**

- ‘Great care should be taken in removing the worm from its attachment’.What does great care mean? Rather it is good if you recommend other techniques.

Are enough details provided of any physical examination and diagnostic tests, treatment given and outcomes?

Yes

Is the case presented with sufficient detail to be useful for other practitioners?

Yes

Is sufficient discussion included of the importance of the findings and their relevance to future understanding of disease processes, diagnosis or treatment?

Partly

Is the background of the case’s history and progression described in sufficient detail?

Yes

Reviewer Expertise:

Gynecology

I confirm that I have read this submission and believe that I have an appropriate level of expertise to confirm that it is of an acceptable scientific standard, however I have significant reservations, as outlined above.

# Response

**Published:** 2021-04-06

**Authors:** Million Abraha

**Affiliations:** University of Bern, Switzerland

Generally there were a minor changes required in the whole manuscript. In the abstract section I removed the word ‘count’ in ‘complete blood cell count’. In the case presentation section the word mother was replaced with patient and the days duration with just 17 days.

 In our practice we usually apply alcohol before we remove the leech from its attachment site.

 Although alcohol can irritate the wounded mucosal surface, it can still help you in removing the leech as it irritate the leech at the same well. To best of our knowledge we still did not find if this contradicting to the idea that no alcohol should be used in removal of leechs.

 The patient was appointed for follow up and the wounded site has healed and there was no fresh blood seen from the previous attachment site. We did not take a photo of the leech because initially there was no idea to publish it as the procedure was being done.

 In discussion section, ‘Great care should be taken in removing the worm from its attachment’. This means we tried to be meticulous with the procedure as it might hurt other part of the vagina during the process.

 I hope i this was explanatory to the questions raised. Thank you everyone for their inputs.
